# Effect of light-curing units in shear bond strength of metallic brackets: an *in vitro* study

**DOI:** 10.1590/S1678-77572010000100012

**Published:** 2010

**Authors:** Luciana Borges RETAMOSO, Niége Michelle Lazzari ONOFRE, Luciane HANN, Ernani Menezes MARCHIORO

**Affiliations:** 1 DDS, PhD student, Master’s degree program in Orthodontics, Pontifical Catholic University of Paraná, Curitiba, PR, Brazil.; 2 DDS, Graduate student, Master’s degree program in Orthodontics, Pontifical Catholic University of Rio Grande do Sul, Porto Alegre, Brazil.; 3 Private Practice, Porto Alegre, RS, Brazil.; 4 DDS, MSc, PhD, Associate Professor of Orthodontics, Pontifical Catholic University of Rio Grande do Sul, Porto Alegre, RS, Brazil.

**Keywords:** Shear bond strength, Halogen light, LED, Brackets

## Abstract

**Objective:**

To determine the influence of the light curing units on the shear bond strength of orthodontic brackets.

**Material and Methods:**

Seventy-two premolars were divided into six groups (n=12): Group I: brackets bonded with Transbond and polymerization with halogen light; Group II: Transbond and LED; Group III: Fuji Ortho and halogen light; Group IV: Fuji Ortho and LED; Group V: Fuji Ortho, without acid and halogen light; Group VI: Fuji Ortho, without acid and LED. The groups were tested to shear strength in a universal testing machine at a crosshead speed of 0.5 mm/min. Data were analyzed statistically by ANOVA and Tukey’s test.

**Results:**

The composite resin presented higher shear bond strength than the resin-modified glass ionomer cement (p<0.05). The halogen light and LED sources produced similar shear bond strength (p>0.05).

**Conclusion:**

The shear bond strength was influenced by the material but not by the light-curing unit. The use of LED reduced the experimental time by approximately 60%, with the same curing efficiency.

## INTRODUCTION

Dentistry has experienced a remarkable progress, starting from the technique of enamel acid etching introduced by Buonocore^6^ (1955). In the same way, the direct bonding of brackets to the teeth revolutionized Orthodontics.

Most orthodontic bonding materials use as the activation mechanism the luminous energy, like quartz-tungsten-halogen (QTH) visible light, xenon light and light-emitting diode (LED)^8,10^. Halogen lamps are the luminous sources most commonly used by orthodontists because they are well known in the literature, have low cost, ease of handling and ease of upkeeping^3^. However, the time spent for the activation of the materials is long and QTH bulbs have a relatively short effective lifetime.

The use of LED technology to polymerize lightactivated dental materials was proposed in the mid-1990s in an attempt to overcome some of the shortcomings of the QTH light-curing units. The use of LED sources for curing of orthodontic materials has been recently introduced and has gained popularity because it has advantages such as a short time to reach material polymerization and longer lifetime^7^, in addition to a stable, efficient, long-lasting output of blue light with little amount of wasted energy and minimum heat generation. As the luminous energy emitted by the diode is in the blue spectral region (450-490 nm)^13,17^, Since a narrow band of light is emitted, there is no need for filter systems.

Composite resins and resin modified glass ionomers (RMGICs) are the most commonly used dental materials for orthodontic boding. RMGICs have some advantages, such as fluoride release, minimal demineralization of the margins of the orthodontic accessories, adhesion to the enamel without need of completely dry field^19,20^. Composite resins have a long working time, ease of handling and no need of mixing, since they are marketed in individual cartridges. The aim of this study was to evaluate in vitro the influence of the materials and light-curing units in the shear bond strength of metallic brackets bonded to human enamel.

## MATERIAL AND METHODS

This research protocol was approved by the Research Ethics Committee of the Pontifical Catholic University of Rio Grande do Sul, Brazil.

**Figure 1:**
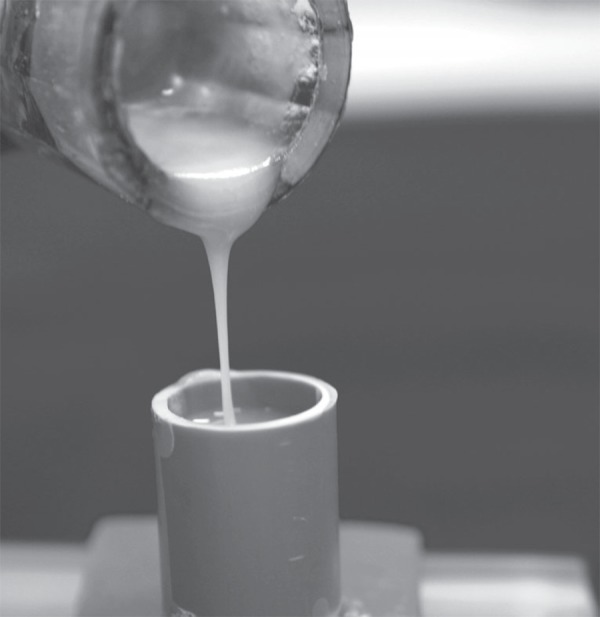
Buccal surface positioned against a glass plate, fastened with wax 7, PVC ring positioned and the acrylic resin flowed

Seventy-two extracted healthy human premolars were selected and stored in distilled water until use. The dental crowns were embedded in standardized PVC (Tigre, Joinville, Santa Catarina, Brazil) rings (20 mm diameter and 20 mm height). The buccal surface was positioned against a glass plate in order to keep most of the flat surface parallel to the ground. In this position, the crown was fixed with 7 wax (Horus, Rio de Janeiro, Rio de Janeiro, Brazil), the PVC ring was correctly positioned and acrylic resin (Jet; Clássico Artigos Odontológicos Ltda., São Paulo, São Paulo, SP, Brazil) was poured into the ring (Figure 1).

The specimens were washed to eliminate the residues originating from the inclusion process and were randomly divided into 6 groups (n=12), according to the bonding material and light-curing unit (Figure 2). The specimens were cleaned with pumice/rubber prophylaxis for 10 s, rinsed with distilled water for 10 s and gently air dried for 20 s at a distance of 50 mm.

**Figure 2:** Groups according to the bonding material and light-curing unit used

**Groups**	**Bonding Material**	**Phosphoric acid**	**Light-curing Units**	**Light intensity**
I	Transbond XT	Yes	Halogen light	450mW/cm^2^
II	Transbond XT	Yes	LED	800mW/cm^2^
III	Fuji Ortho	Yes	Halogen light	450mW/cm^2^
IV	Fuji Ortho	Yes	LED	800mW/cm^2^
V	Fuji Ortho	No	Halogen light	450mW/cm^2^
VI	Fuji Ortho	No	LED	800mW/cm^2^

In GI, the enamel surface was etched with 37% phosphoric acid (Dentalville, Joinville, Santa Catarina, Brazil) for 15 s, rinsed with distilled water for 10 s, air-dried for 10 s at a 5 cm distance and coated with Transbond XT^®^ primer (3M/ Unitek, Monrovia, CA, USA). Then, each stainless steel premolar bracket (Victory Series; 3M/ Unitek, Monrovia, CA, USA) received a layer of Transbond XT^®^ adhesive resin (3M/Unitek, Monrovia, CA, USA) on its base and was positioned on the buccal enamel surface and pressed with 400 kgf, measured by a dynamometer (Morelli, Campinas, São Paulo, Brazil). Excess adhesive was removed from around the bracket base and the material was light cured by positioning the light guide tip of a halogen lamp (Ortholux XT^®^ lamp, 3M/Unitek, Monrovia, CA, USA) on each interproximal side for 10 s.

In GII, after bonding as described in GI, Transbond XT adhesive resin (3M/Unitek, Monrovia, CA, USA) was light cured by positioning the light guide tip of a LED source (Ortholux LED^®^ , 3M/Unitek, Monrovia, CA, USA) on the mesial and distal sides for 7.5 s.

In GIII, the enamel surface was etched with 37% phosphoric acid (Dentalville, Joinville, Santa Catarina, Brazil) for 30 s, rinsed with distilled water for 10 s and air-dried for 10 s at a 5 cm distance. Then, each bracket received a layer of Fuji Ortho LC^®^ (GC America Inc., Chicago, IL, USA) on its base and was positioned on the buccal enamel surface and pressed with 400 kgf, measured by a dynamometer (Morelli, Campinas, São Paulo, Brazil). Excess adhesive was removed from around the bracket base and the material was light cured by positioning the light guide tip of a halogen lamp (Ortholux XT^®^ lamp, 3M/ Unitek, Monrovia, CA, USA) on each interproximal side for 10 s.

In GIV, after bonding as described in GIII, Fuji Ortho LC^®^ (GC America Inc., Chicago, IL, USA) was light cured by positioning the light guide tip of a LED source (Ortholux LED^®^, 3M/Unitek, Monrovia, CA, USA) on the mesial and distal sides for 7.5 s.

In GV, Fuji Ortho LC^®^ (GC America Inc., Chicago, IL, USA) was applied on the bracket base, positioned on the buccal enamel surface and pressed with 400 kgf, measured by a dynamometer (Morelli, Campinas, São Paulo, Brazil). Excess adhesive was removed from around the bracket base and the material was light cured by positioning the light guide tip of a halogen lamp (Ortholux XT^®^ lamp; 3M/Unitek, Monrovia, CA, USA) on each interproximal side for 10 s.

In GVI, after bonding as described in GV, Fuji Ortho LC^®^ (GC America Inc., Chicago IL, USA) was light cured by positioning the light guide tip of a LED source (Ortholux LED^®^, 3M/Unitek, Monrovia, CA, USA) on each interproximal side.

The light intensity emitted by the halogen light and LED light-curing units was measured by digital and analogical radiometers (Demetron, Kerr, CA, USA), respectively. The bonding materials were used according to the manufacturers’ instructions.

**Figure 3:**
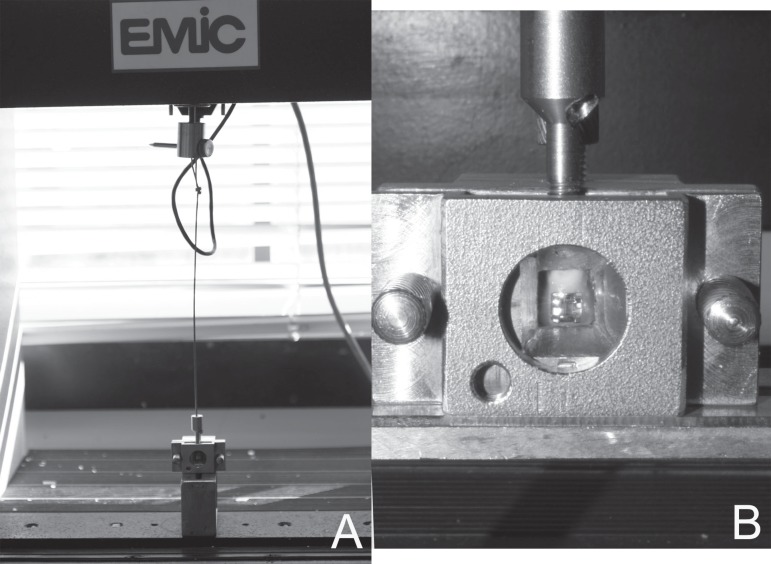
Specimens (A and B) stressed in a universal testing machine at a crosshead speed of 0.5 mm/min

After bonding, all specimens were stored in distilled water at 37ºC for 24 h and then tested in a shear mode on a universal testing machine (EMIC DL 2000, São José dos Pinhais, Paraná, Brazil). Specimens were secured in the lower jaw of the machine so that the bonded bracket base was perpendicular to the shearing force direction. Specimens were stressed in an occlusogingival direction at a crosshead speed of 0.5 mm/min (Figure 3). The maximum load necessary to debond or initiate bracket fracture was recorded in N and then converted into MPa as a ratio of N to bracket surface area. The bracket base area was measured (mean 14.28 mm^2^) using digital caliper accurate to 0.01 mm (Electronic Caliper 227; Starret, Itu, São Paulo, Brazil).

After bracket debonding, the adhesive remnant index (ARI) was verified with an optical microscope at ×40 magnification^2^. The failure modes were classified according to 4-point scoring system: 0 = no adhesive remaining on the tooth; 1 = less than half of the adhesive remaining on the tooth; 2 = more than half of the adhesive remaining on the tooth; and 3 = all adhesive remaining on the tooth, with an impression of the bracket mesh.

**Figure 4:**
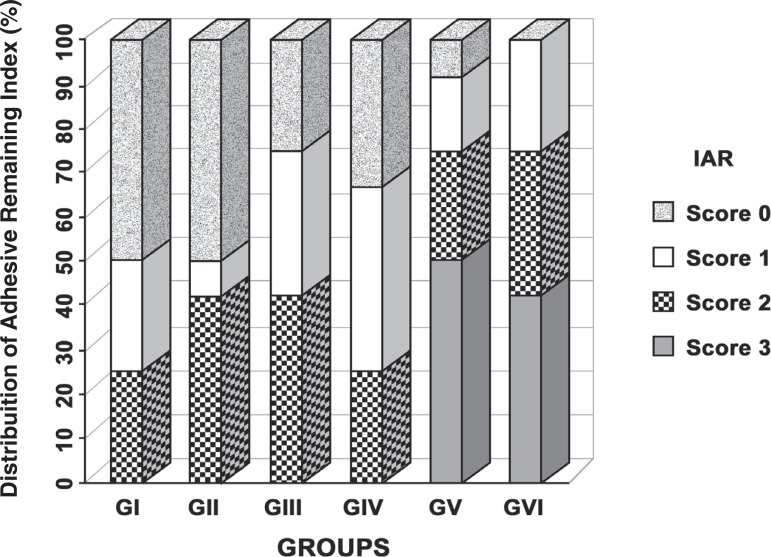
Adhesive Remaining Index (ARI) distribution in the groups

Data were analyzed using the Statistical Package for the Social Science 13.0 for Windows (SPSS Inc., Chicago, IL, USA). Kolmogorov-Smirnov and Levene tests were used to verify normality and homogeneity, respectively, with the significance level set at 0.05. Two-way ANOVA was used to verify intergroup differences because the variables demonstrated normal distribution and homogeneity, followed by Tukey’s multiple comparison test. The ARI data were analyzed by the chi-square test among the groups. The results were evaluated within a 95% confidence interval (Figure 4).

## RESULTS

There was no statistically significant difference (p>0.05) among the groups when the influence of the light-curing units was considered. However, the results were significantly influenced (p<0.05) by the material used for orthodontic bonding (Group I and II> Group III, IV, V and VI). Enamel etching influenced the shear bond strength in groups, great part of the adhesive remained on the group light cured with LED (Group III and IV> Group VI) (Table 1).

**Table 1:** Description statistics for shear bond strenght

**Groups**	**n**	**Material**	**Light Curing Unit**	**Mean**	**Standard deviation**
I	12	Transbond XT	Halogen light	14.06^A^	3.75
II	12	Transbond XT	Halogen LED	13.08^A^	2.54
III	12	Fuji Ortho	Halogen light	7.85^B^	2.36
IV	12	Fuji Ortho	Halogen LED	5.49^B^	1.95
V	12	Fuji Ortho	Halogen light	3.83^BC^	0.92
VI	12	Fuji Ortho	Halogen LED	2.96^C^	0.29

Different letters in mean column indicated statistical difference to Tukey HSD (p<0.05)

The ARI scores were distributed as shown in Figure 4. Most specimens of Groups V and VI failed at the enamel/adhesive interface, which means that the whole adhesive layer remained on the bracket. In the specimens of the other 4 groups, great part of the adhesive remained on the enamel, with the impression of the bracket base on the remainder. When the ARI is analyzed comparing the materials, failure at the adhesive/ bracket interface (score 3) was more common in the specimens of the Groups I and II, while in the specimens of Groups III and IV there was an even distribution among scores 2 and 3, though without statistical significance (p>0.05).

## DISCUSSION

Adhesion in Orthodontics is considerably less critical than adhesion in Restorative Dentistry because it involves only the attachment of orthodontic components to enamel. Bonding to dentin, which is routinely seen in Restorative Dentistry, is far more challenging because dentin is a composite of apatite crystals embedded in a collagen matrix, with dentinal tubules that communicate with the pulp and contain interstitial fluid. In addition to the mineral phase, bonding to dentin basically relies on the organic phase of this substrate^1^. In addition to this, the following factors also contribute to make adhesion to enamel less complex: bonding in orthodontic needs approximately 2 year-old durability; enamel acid etching is not capable of causing pulpal damage; color alteration is not critical; and problems with abrasion are not significant^18^.

However, inadequate polymerization of dental composites has been associated with inferior physical properties, retention failures, higher solubility, and adverse pulpal responses because of unpolymerized residual monomers. Therefore, the capacity of light-curing units to deliver sufficient light at appropriate absorption maximum levels for the respective photoinitiator systems is crucial to optimize the physical properties of light-activated dental materials^9^.

In Orthodontics, the most important of these factors is whether the adhesive composite has reached a level of polymerization that will adequately retain brackets to teeth when orthodontic forces are applied. Direct bonding in Orthodontics using halogen light sources is a common procedure in the routine of orthodontists, but the use of other light sources, like LED units, has also become a usual and acceptable practice for bracket bonding. Clinical success should be associated with a shorter time for bonding procedures. Taking in view some advantages and differences among halogen light and diode curing units, the present in vitro study compared the shear bond strength of brackets bonded with different materials (RMGIC versus composite resin) and polymerized with different light sources (LED versus halogen light).

Regarding the light curing source, there was no significant difference in the shear bond strength for any of the materials evaluated in this study, which is in agreement with the results of previous investigations^5,9^. However, those studies used similar curing times for the halogen and LED source, while in the present study a shorter curing time was used for the LED unit, demonstrating a great efficiency for polymerizing orthodontic bonding materials. It may be speculated that this difference is due to the fact that LED emission spectrum is close to the maximum absorption peak of camphorquinone. According to Mills et al.^15^ (1999), only a small portion of the emission spectrum of halogen lights is actually used for activating photoinitiator molecules, while LED units are more efficient in delivering usable light to activate the camphorquinone.

Usumez, et al.^22^ (2004) found lower shear bond strength when LED was used with a shorter curing time than halogen light, but Swanson, et al.^21^ (2004) reported clinically acceptable shear bond strength for brackets bonded with LED using 10-s curing time. Jandt, et al.^12^ (2000) reported that composite materials had higher depth of cure when photoactivated with halogen light compared to LED. However, both halogen and LED units cured the composites deeper than required by ISO 4049 standard and the manufacturer^12^. In orthodontics, a high depth of cure is not necessary because orthodontic materials are used in thin layers.

During the choice of the bonding material, some factors should be taken in consideration: resistance, longevity, and removal of excesses without damaging tooth surface. These factors can be evaluated in vitro by assessing the shear bond strength and ARI^16^ values and further transposed to the clinical practice.

Analyzing the bonding material, it was noticed that the composite resin presented effective adhesion to the dental enamel (between 13 and 14 MPa), while the RMGIC presented lower shear bond strength (between 5 and 8 MPa). Similar results were obtained by Bishara, et al.^4^ (1999) and Lippitz, et al.^14^ (1998). However, a high shear bond strength is not always a desirable characteristic because brackets frequently need to be removed during the orthodontic treatment and a high bond strength can produce damage to the dental enamel^1,11^.

Another important finding of this work is the importance of the pretreatment of the dental surface. No enamel etching prior to the use of the RMGIC for bracket attachment reduced the bond strength to levels that are not clinically acceptable (2 to 4 MPa). This result agrees with those Reynolds^18^ (1975).

The analysis of ARI indicated that in most specimens of Groups I, II, III and IV the material remained adhered to the dental surface after the debonding of the accessories, independent of the light-curing unit used, suggesting that the weakest adhesion occurs between the metallic bracket and the bonding material (RMGIC and composite resin). The adhesion of the orthodontic metallic accessory to the acid-etched enamel seems indicated since none of the groups presented score 0 in ARI. However, the non conditioning of the enamel provided a different pattern, since ARI varied between 0 and 1, indicating a weak adhesion between enamel and the RMGIC.

Analyzing bracket debonding, it is desirable that the failure occurs between the bracket and the adhesive or at the adhesive interface. Failure between adhesive and enamel can create enamel fractures or cause irregularities^4^. In that way, neither the composite resin (Transbond XT) nor the RMGIC (Fuji Ortho LC) polymerized by any one of the light sources used in this study would cause damage to tooth surface because most failures occurred between bracket and adhesive, reducing the chances of enamel fracture. On the other hand, there was greater difficulty in removing materials adhesives excesses, which is consistent with the findings of Dunn and Taloumis^9^ (2002) which found ARI scores around 2 and 3.

It is important to point out that the main goal of this study was to assess the shear bond strength of metallic brackets bonded to enamel with different materials polymerized with a LED unit using a shorter curing time than the halogen light. This means, shorter clinical time and greater comfort to the patients and orthodontists.

## CONCLUSIONS

The following conclusions may be drawn from the obtained results: 1. The light-curing units (halogen or LED) did not influence the shear bond strength of orthodontic brackets to enamel, but the orthodontic material influence bracket adhesion; 2. No acid conditioning of enamel influenced the bond strength of brackets bonded with the RMGIC (Fuji Ortho LC), resulting in values that are not acceptable for clinical conditions; 3. The use of LED reduced the experimental time by approximately 60%, with the same curing efficiency.
